# Green Recycling of Carbon/Carbon Composites by Solid-State Shear Milling Technology as a Polyamide Multi-Functional Modifier

**DOI:** 10.3390/polym16212962

**Published:** 2024-10-23

**Authors:** Qianyue Tan, Shuangxin Lai, Liang Xue, Haiping Liu, Shibing Bai

**Affiliations:** 1 State Key Lab of Polymer Materials and Engineering, Sichuan University, Chengdu 610065, China; tqianyue@163.com (Q.T.); 18180570010@163.com (S.L.); 2 Xi’an Yuanchuang Aviation Technology Co., Ltd., Xi’an 710061, China; x652418@163.com (L.X.); llhhpp198123@126.com (H.L.)

**Keywords:** recycle, C/C composites, PA, reinforcing, heat deflection, wear resistance

## Abstract

Carbon/carbon (C/C) composite materials are widely used in aerospace, the military and nuclear energy. The outstanding mechanical qualities of C/C composites mean that they are difficult to crush and recycle using traditional technology. The current recycling methods primarily involve stacking and landfill disposal. Therefore, achieving efficient and environmentally friendly recycling of carbon/carbon (C/C) composites is an urgent and challenging issue. In this work, we reported a simple high-value recycling approach for carbon–carbon frictional composite material (CFCM). The solid-state shear milling (S^3^M) technology is employed to achieve ultrafine milling of carbon matrices in carbon/carbon (C/C) composite materials while preserving carbon fibers. By this means, carbon fibers and the carbon matrix were mainly split, and the prepared composite powder had combined functionalities of conductivity, thermal conductivity, reinforcement, and wear resistance. The experimental results showed that the tensile strength of the material increased from 64.35 MPa to 72.79 MPa after being compounded with PA6, and the thermal conductivity increased from 0.211 W/mK to 0.611 W/mK. The friction coefficient was reduced from 0.51 to 0.36, a reduction of 25.4%, and the heat deflection temperature was increased from 47.2 °C to 108.2 °C. The S^3^M technique proposed in this work is an efficient, high-value, and scalable recycling strategy for CFCM, which can be used to produce value-added products and has great application prospects.

## 1. Introduction

Carbon/carbon (C/C) composites are solid materials constructed of carbon fibers and textiles as reinforcing agents, carbonaceous materials such as thermally decomposed carbon or pitch carbon as the matrix, and created through specified processing methods [1,2,3]. The preparation methods of C/C composites can be mainly divided into chemical vapor deposition (CVD) [4] and liquid phase impregnation (LPI) [5] due to the different densification processes. CVD has the advantage of tight bonding between the matrix carbon and the fiber and adjustable structure, making it the preferred method for preparing high-performance C/C composites [6,7]. However, the price of C/C composites is significantly influenced by the slowness of the CVD process and the lengthy production cycle, and there is a significant amount of waste during the manufacturing process [8]. C/C composites have the characteristics of low density, high thermal conductivity, low coefficient of thermal expansion, and excellent mechanical properties, and are widely used in aerospace, the military, nuclear energy, and many other fields [9,10,11,12]. Their unique properties of high strength and toughness, high resistance to thermal shock, low coefficient of thermal expansion, and sufficient braking performance make them the best material for manufacturing brakes of aircrafts and other high-weight vehicles, with great development potential [13,14,15,16]. In 2023, China produced approximately 550 tons of C/C composite materials, which was a 17.02% increase from the previous year. Recycling C/C composites is challenging due to the inability to chemically or thermally split the carbon fibers and matrix carbon using current technologies. This material is currently disposed of by landfill as a foundation material, resulting in a great waste of resources. So, there is an urgent need to develop a new process for recycling C/C composite materials.

**Figure 1:**
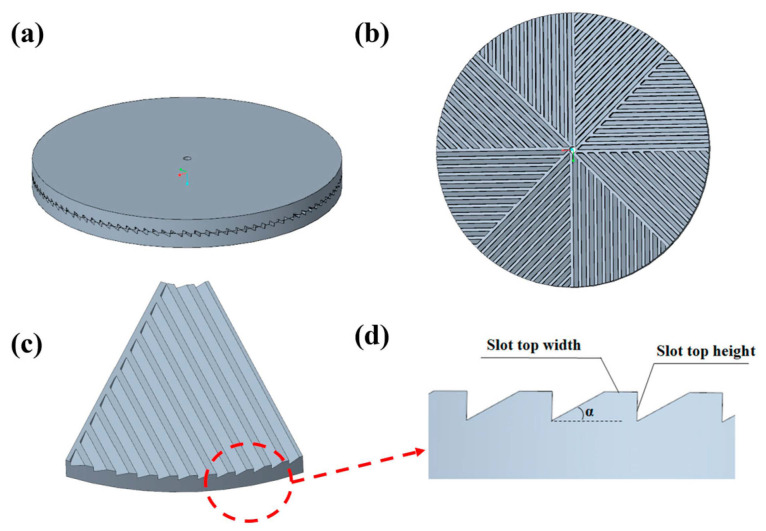
(**a**) Side view of mill pan; (**b**) complete and (**c**,**d**) detailed structure of mill pan surface.

Our research group has previously reported solid shear milling (S^3^M) technology, which acts as a three-dimensional scissor during milling by utilizing its distinctive structure (Figure 1) [14,17,18]. This technology applies a powerful three-dimensional shearing force to the material and exhibits multi-functionality such as comminution, dispersion, mixing, and activation [19]. In response to the difficulty of achieving the split of carbon fibers and carbon matrix in C/C composites, a new strategy for recycling C/C composites was proposed, producing a multifunctional composite powder with thermal, electrical, reinforcing, and wear-resistant properties.

Polyamide 6 (PA6) is characterized by high strength, self-lubricating properties, good damping properties, corrosion resistance, resistance to ultraviolet and gamma radiation, and a relatively simple and economical manufacturing process, making it widely used in wear-resistant materials for applications such as aircraft, automobiles, and machinery [20,21,22,23]. Over the past few decades, numerous researchers have enhanced the mechanical, thermal, and wear resistance characteristics of PA6 through carbon fibers [24,25,26,27,28]. However, these studies tend to use commercially available carbon fibers, ignoring the great recycling value of carbon fibers in C/C composites. In this paper, the recycling and utilization of carbon fibers in C/C composites have been achieved for the first time by using S^3^M technology. The recycled C/C composite powder was added to PA6, significantly improving its mechanical properties, thermal conductivity, friction properties, and thermal stability. This proposed method can solve the problem of environmental pollution and realize the production of high-value composite materials.

## 2. Materials and Methods

### 2.1. Materials

The carbon–carbon (C/C) frictional composite material (CFCM) used in this study was provided by Xi’an Aviation Brake Technology Co., Ltd. (Xi’an, China). Polyamide 6 (PA6) (B30S), with a melting temperature of 222 ℃ and a density of 1.14 g/cm^3^, was purchased from LANXESS (Cologne, Germany).

### 2.2. Preparation of Composite Materials

Firstly, CFCM must be crushed by a powerful crusher, and then further milled by the S^3^M equipment invented by our research group. The core part of the S^3^M equipment is the inlaid pans made of wear-resistant metal. In a cycle of rotation, the upper and lower milling surface between the two sides of the angle gradually reduced to 0. During the milling process, the S^3^M equipment acts as a three-dimensional scissor, generating a powerful shearing force and pressure, which can achieve crushing, dispersion, mixing, and mechanical chemical modification of the material [29]. The pressure between the milling pans of the S^3^M equipment is 3 MPa, the rotational speed of the milling pans is 100 rpm, and the cooling water temperature is 25 °C. CFCM powders generated by different milling cycles are denoted as CFCM-n. Materials enter the equipment from the feeding port and leave through the discharging port after completing milling, which is called a milling cycle.

The PA6 was dried in an oven at 80 °C for 4 h and subsequently at 120 °C for 5 h. The CFCM powder was dried in a vacuum drying oven at 80 °C for 5 h and then mixed uniformly with PA6. The proportion of CFCM powder in the composite was 30 wt%. The mixed material was extruded through a twin-screw extruder with the extruder temperature sequentially set as 165 °C, 190 °C, 220 °C, 250 °C, 250 °C, 250 °C, 250 °C, 250 °C, 245 °C, and 240 °C and the screw speed kept at 300 rpm. The samples were prepared by injection molding, with the temperature control in the hopper to the nozzle set at 165 °C, 190 °C, 250 °C, and 250 °C.

### 2.3. Characterization

SEM (SEM-450, FEI Instrument Co., Hillsboro, OR, USA) was used to observe the fiber debonding, aspect ratio, dispersion, and wear surface morphology of the CFCM. The elemental composition of the CFCM was analyzed using energy-dispersive X-ray (EDX) elemental analysis instrument. The cross-section of the composite material needed to be fractured in liquid nitrogen and all samples were pre-coated with gold before testing.

The thermal conductivity of the samples was measured using a thermal conductivity meter (Hot Disk TPS2500S, Hot Disk AB, Gothenburg, Sweden), and the sensor was placed between two identical samples. The samples were tightened to ensure good contact with the sensor.

The electrical properties of the CFCM powder before and after different grinding cycles were compared using a four-probe method. The electrical conductivity was measured at 15 pressure points in the range of 2-30 MPa and compared at the same pressure.

The melting and crystallization behavior of the composite material was studied using a differential scanning calorimeter (TA-Q20, Waters Technology (Shanghai) Co., Ltd., Shanghai, China). The temperature range was 40–250 °C, the heating (cooling) rate was 10 °C/min, and the sample was kept at 200 °C for 5 min to eliminate the thermal history. The DSC curve of the CFCM/PA6 composite material was measured in a nitrogen atmosphere.

The thermal stability of the composite material in air and nitrogen atmospheres was determined using a thermogravimetric analyzer (TA-Q50, Waters Technology (Shanghai) Co., Ltd., Shanghai, China), with a temperature range of 40–700 °C and a heating rate of 10 °C/min.

The wear test was conducted using the Bueker UMT-3 Tribometer (Bellerica, MA, USA) with a GCr15 stainless steel ball sliding over the sample surface. A 4.75 mm diameter stainless steel ball was installed on a support frame connected to a three-dimensional force sensor, and the sample was tested using a ball-on-plate apparatus. The normal load applied was 10 N, and the wear test was conducted for 6000 cycles in dry conditions.

The tensile and bending properties of the material were tested using a universal testing machine (Instron 5576 Massachusetts, Instron GmbH, Hesse, Germany) according to the ASTM Standard D638-10 [30] and ASTM Standard D790-10 standards [31]. Impact testing was conducted using a plastic pendulum impact tester (PIT 501J, Shenzhen Wantest Equipment Co., Ltd., Shenzhen, China), and the impact properties of the material were tested according to the ASTM D6110-2010 standard [32].

The thermal deformation temperature of the composite material was tested using a thermal deformation temperature tester (HDV2, Atlas, Sweden) with a load of 1.8 MPa.

## 3. Results and Discussion

### 3.1. The Crushing Mechanism and Disposal Procedure of S^3^M

The manufacturing process we designed includes the preparation of CFCM and the split of carbon fibers from the carbon matrix using the S^3^M technology while maintaining a high degree of the carbon fiber aspect ratio. In this work, CFCM was successfully recycled through the S^3^M process.

**Figure 2:**
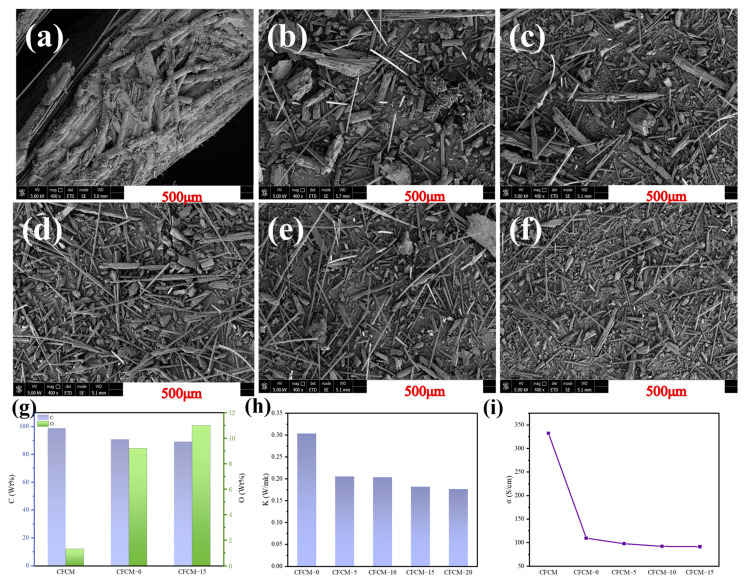
SEM images of (**a**) as-received CFCM; (**b**) CFCM-0; (**c**) CFCM-5; (**d**) CFCM-10; (**e**) CFCM-15; (**f**) CFCM-20; (**g**) element composition of CFCM by EDS; (**h**) thermal conductivity of CFCM; (**i**) electrical conductivity of CFCM.

Scanning electron microscopy (SEM) was employed to observe and assess the morphological changes in CFCM during the S^3^M process in this study. As shown in Figure 2a–f, the carbon matrix uniformly surrounds the carbon fibers in the CFCM; after crushing, the CFCM particles have larger diameters, and the carbon fiber bundles still have a large amount of the carbon matrix wrapped on their surfaces and inside, making it difficult to split up. During the milling process, CFCM is subjected to powerful three-dimensional shearing, and S^3^M can effectively split the carbon fibers from the carbon matrix while maintaining their aspect ratio to the greatest extent. The carbon fiber bundles gradually break down as the milling cycle increases; the fiber average length reduces; the surface roughness rises; certain narrow grooves are longitudinally scattered along the fibers. When the milling cycle increases to 20, effective split of the carbon fibers from the carbon matrix can be achieved, and the carbon fibers are distributed as single fibers. 

The EDS results of CFCM shown in Figure 2g indicate that both crushing and milling can increase the oxygen content on the surface of CFCM, providing potential value for the further application of CFCM. The tensile strength and modulus of carbon fiber are very high, but the fiber is not resistant to shear. As an example, for the tensile strength of T300, its tensile strength can reach 230 GPa, while the shear strength is only 10.9 GPa [33]. As a result, carbon fibers are more susceptible to fracture when subjected to strong shear strength. In the milling process of S^3^M, as the rotating motion of the moving milling pan is applied, the angle between the upper and lower milling surfaces gradually decreases to zero, resulting in strong compression and three-dimensional shear on the material [34,35]. The maximum force applied to the material unit by milling is related to the spindle torque, which can be calculated by the following formula [36]:

$$F=\frac{M}{d}$$

In the equation, M represents the torque, d is the distance from the force center to the torque center, and *F* is the force exerted on the force point when the torque is applied.

The finite element analysis results of S^3^M carried out in recent research work show that the maximum stress in most material units appears in the contact area between the milling pan and the material, and the position is close to the junction of the upper and lower milling pan. The stress can reach up to 6.3 × 10^4^ MPa, which is much higher than the shear strength of carbon fiber, thus achieving the fracture of carbon fiber [37,38].

The strong force exerted by the S^3^M machine on the C/C composites is effective in separating the carbon fibers from the carbon matrix, but the carbon fibers are less resistant to shear, so the milling process leads to a reduction in the length of the carbon fibers. When the length of the carbon fiber decreases, the electron transfer path between the carbon atoms becomes shorter, and the contact resistance between the short fibers increases, thus reducing the overall electrical conductivity. The thermal conductivity of carbon fibers is also related to their length. When the length of carbon fibers decreases, the continuity of the thermal conductivity network is affected, resulting in a decrease in heat transfer efficiency. In addition, gaps and irregular arrangements between short fibers also increase thermal resistance, further reducing thermal conductivity. The fact that the fiber length decreases with the increase in the number of milling cycles helps to explain why the thermal conductivity of CFCM decreased from 0.30 W/mk to 0.21 W/m and the electrical conductivity decreased from 332.31 S/cm to 109.38 S/cm.

### 3.2. Crystallization and Thermal Stability of PA6/CFCM Composites

**Figure 3:**
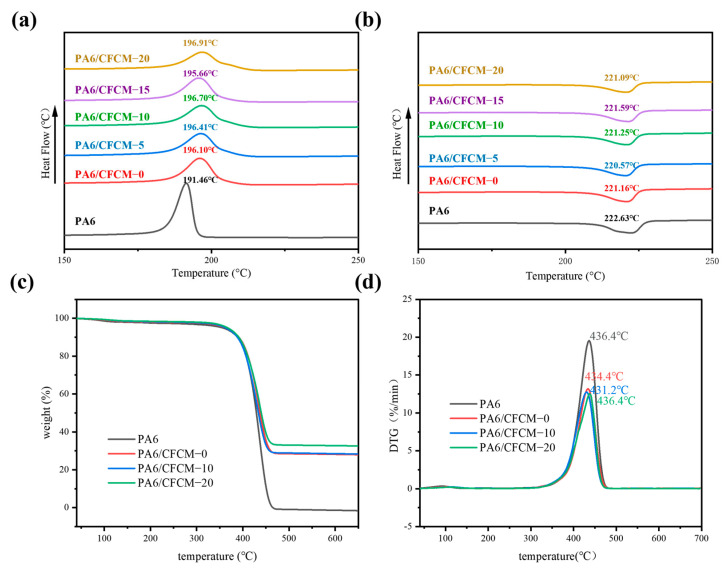
Chemical characterization of PA6/CFCM composites: DSC crystallization curve (**a**); DSC heating curve (**b**); TG (**c**,**d**).

The DSC curves of the obtained CFCM/PA6 composite materials are shown in Figure 3a,b. The crystallization temperature Tc, melting temperature Tm, glass transition temperature Tg, and crystallinity χc are all listed in Table 1. The results show that compared with pure PA6, adding CFCM increases the Tc of the composite material by approximately 5 °C. The CFCM powder utilized in the composite material demonstrates the capacity for heterogeneous nucleation, accelerating nucleation, and increasing the crystallization temperature. In comparison to the crystallinity of pure PA6, the introduction of CFCM fibers can enhance the crystallinity of the composite material, which is beneficial for the mechanical properties of the composite material. However, the glass transition temperature of the composite material decreases as a result of the poor interfacial bonding ability of the CFCM/PA6 composite material [39].

Figure 3c,d show the TGA results of PA6 and CFCM/PA6 composite materials, tested at temperatures ranging from 40 to 700 °C. According to the TGA curve analysis, the largest mass loss occurred between 355 and 465 °C, and the thermal stability improvement was not significant. During the test, PA6 was almost completely consumed, and the residual weight of the composite with 30 wt% CFCM was consistent with the weight percentage of CFCM in the particles, but the residual carbon increased when the milling cycle was 20, implying that the addition of CFCM promoted the formation of carbon.

### 3.3. Morphology and Friction Properties of the Composites

**Figure 4:**
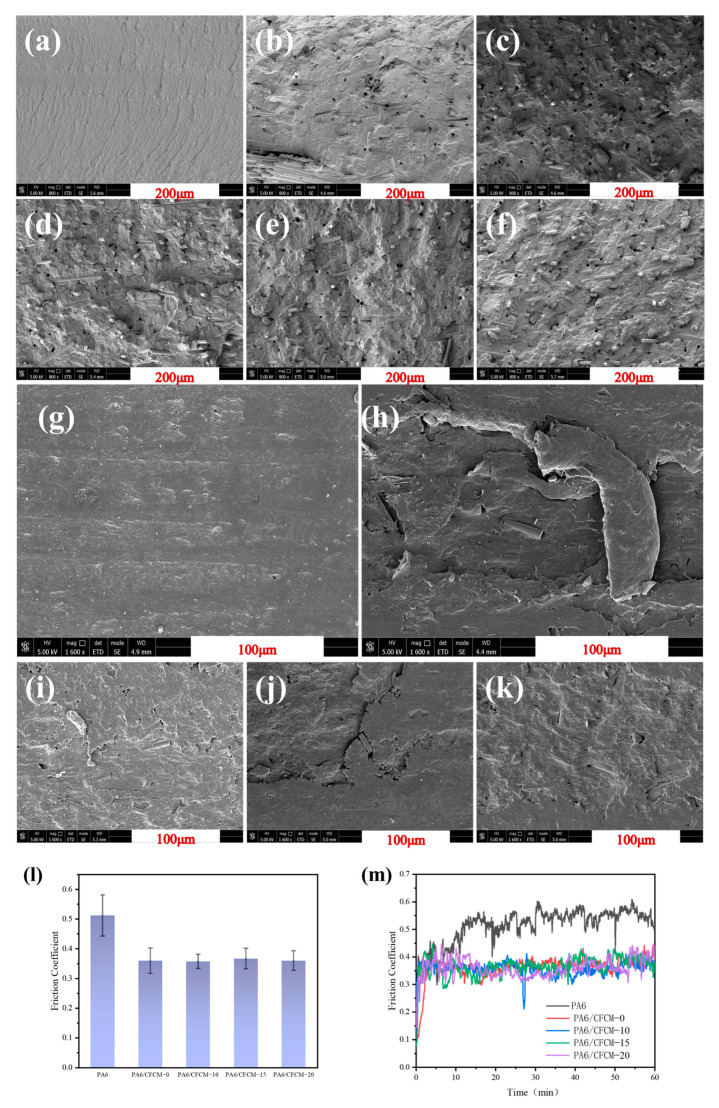
SEM images of (**a**) PA6; (**b**) PA6/CFCM-0; (**c**) PA6/CFCM-5; (**d**) PA6/CFCM-10; (**e**) PA6/CFCM-15; (**f**) PA6/CFCM-20; worn surfaces of PA6/CFCM composites: (**g**) PA6; (**h**) PA6/CFCM-0; (**i**) PA6/CFCM-10; (**j**) PA6/CFCM-15; (**k**) PA6/CFCM-20; (**l**,**m**) the friction behavior of PA6/CFCM composites.

The distribution, morphology, and interfacial properties of carbon fiber in the PA6 matrix can directly affect the thermal conductivity, wear resistance, and mechanical properties of the composite material. Figure 4a–f show the SEM morphology of the cross-section of the PA6/CFCM composite material. As the milling cycle increases, the carbon fiber bundles gradually disappear, and the carbon fibers can be uniformly dispersed in the PA6 matrix. The voids around the carbon fibers indicate that the compatibility between the carbon fibers and the matrix is poor and the interfacial adhesion is relatively weak. The position of the fiber inserted into the matrix and the circular holes left after the fiber was pulled out can be observed.

The coefficient of friction (COF) of pure PA6 and CFCM/PA6 composites is shown in Figure 4l,m. The transient COF fluctuates significantly with the sliding distance, which is caused by the wear debris generated during the friction process. Compared to pure PA6, the COF of CFCM/PA6 composites decreased from 0.51 to 0.36, representing a reduction of approximately 25.4%. This is because CFCM improves the deformation and anti-plowing properties of the composite material as a reinforcing agent.

The surface morphology of the CFCM/PA6 composite material was analyzed using a scanning electron microscope and is shown in Figure 4g–k. The wear marks generated by the steel ball friction were visible on the surface of PA6, which was due to the poor self-lubricating performance of PA6. The primary cause of wear during the friction process was the erosive fatigue damage caused by the hard counterpart [21]. From the surface morphology of the CFCM/PA6 composites shown above, a small amount of carbon fiber was exposed on the friction surface, indicating that those fibers bore most of the load, which was beneficial for reducing the wear rate. Cracks can be observed at the fiber–matrix interface under the action of mechanical force. The wear debris of the PA6 matrix was deformed and transferred back to the wear surface. These fragments filled the cracks/cavities on the wear surface to some extent, repairing the fiber/matrix interface and forming a friction surface, thereby reducing the coefficient of friction [26].

### 3.4. Mechanical Properties of Composites

**Figure 5:**
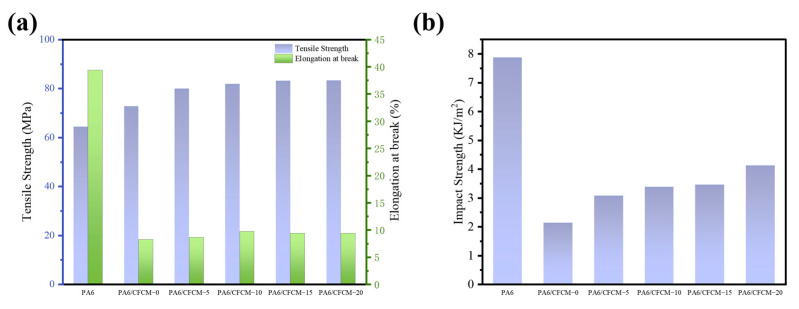
Mechanical properties of CFCM/PA6 composites: (**a**) tensile properties; (**b**) impact strength.

Figure 5a,b illustrate the results of the tensile and impact tests, which demonstrate that the tensile elongation rate decreases from 39.37% to 8.22%, the impact strength decreases from 7.87 KJ/m^2^ to 2.13 KJ/m^2^, and the tensile strength increases from 64.35 MPa to 72.79 MPa. When crushed CFCM was compounded with PA6 at a ratio of 30 wt%. The elongation at break and impact strength experiences a sharp decline as a result of the poor interface bonding between the fibers and matrix [40]. The impact strength of the composite material decreases due to the high number of stress concentration sites at the fiber ends. The tensile strength and impact strength of the composite material increase with milling cycles; after 20 milling cycles, the tensile strength and impact strength of the CFCM fiber composite material are 14.49% and 93.43%, respectively, which are higher than the values reported for composite materials, in which CFCM was only treated by crushing. As the milling cycle increases, the degree of CFCM fiber delamination and surface roughness improve, increasing the contact area between individual fibers and the PA6 matrix, leading to an increase in the contact area of individual fibers with the PA6 matrix. This result is beneficial for the stress transfer between the filler and the matrix under external force and then improves the tensile strength and impact strength of the composite material [41].

### 3.5. The Thermal Conductivity Coefficient and Thermal Deformation Temperature of Composites

**Figure 6:**
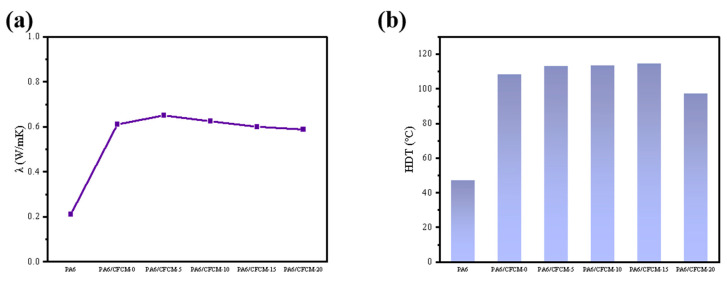
(**a**) Conductivity of CFCM/PA6 composites; (**b**) HDT of CFCM/PA6 composites.

The thermal conductivity of CFCM/PA6 composite material is shown in Figure 6a. The thermal conductivity increased from 0.211 W/mK to 0.611 W/mK after adding CFCM. The improvement of the thermal conductivity can effectively reduce the maximum temperature of the contact surface during high-speed friction, avoiding the aggravation of wear caused by surface softening [42]. The thermal conductivity of the composites experienced a slight decline as milling cycles increased. This can be attributed to the decrease in the length of carbon fibers, which subsequently destroyed the thermal conductivity potential of the composites.

The heat deflection temperature (HDT) is one of the main indicators for evaluating the heat resistance of plastics. Improving HDT is beneficial for the recycling use of CFCM/PA6 composites. The impact of the CFCM milling cycle on the HDT of the CFCM/PA6 composite material is illustrated in Figure 6b. The HDT of PA6 is approximately 47.2 °C, which increased to 108.2 °C after adding 30 wt% CFCM. HDT gradually increased to 114.6 °C as the milling cycle increased, and then decreased to 97.3 °C after 20 milling cycles. In comparison to pure PA6, HDT increased by up to 67.4 °C. This is due to the fiber reinforcement effect. The carbon fiber added to the CFCM can resist the three-point bending load applied during the measurement [43]. As the number of milling cycles increases, HDT of the composite material initially increases and subsequently decreases. This is because the reinforcing fibers with a higher aspect ratio can effectively transmit the applied load to the surrounding fibers through the matrix. After 20 milling cycles, although effective delamination of the carbon fibers and carbon matrix in the CFCM was achieved, the fiber length was reduced, resulting in a decrease in HDT [44].

## 4. Conclusions

The S^3^M technology was employed to recycle carbon/carbon composites in this study. This method is scalable, straightforward, and environmentally friendly. During the milling process, under the action of a strong shearing force and pressure, the carbon fibers and carbon matrix in the C/C composites gradually peel off. After 20 milling cycles, the carbon fibers and matrix completely split. Furthermore, after milling treatment, the surface roughness of carbon fiber increases, and some narrow grooves are distributed longitudinally along the fiber, which is conducive to the interaction between carbon fiber and polymer matrix, thereby improving the interaction between matrix and fibers. The scanning electron microscope analysis showed that the carbon fibers and carbon particles generated by the milling of CFCM were well dispersed in PA6, which improved the mechanical properties of the composites. After 20 milling cycles, the tensile strength and impact strength of the composites made from the milled CFCM were 14.49% and 93.43% higher, respectively, compared to the composite material made from the crushed CFCM. In addition, the thermal conductivity increased from 0.211 W/mK to 0.611 W/mK, and the heat distortion temperature increased by 67.4 °C in comparison to pure PA6. The friction coefficient decreased by 25.4%. Consequently, the S^3^M technology offers a novel method of recycling.

## Figures and Tables

**Table 1:** DSC results of PA6/CFCM composites.

	PA6	PA6/CFCM-0	PA6/CFCM-5	PA6/CFCM-10	PA6/CFCM-15	PA6/CFCM-20
**ΔHm (J/g)**	58.01	57.46	57.14	57.15	56.94	51.35
**ΔHc (J/g)**	74.35	61.01	62.08	56.59	57.75	48.45
**χc %**	30.53	43.20	42.96	42.97	42.81	38.61
**Tg (°C)**	106.31	104.27	105.60	105.39	104.27	104.01
**Tm (°C)**	222.63	221.16	220.57	221.25	221.59	221.09
**Tc (°C)**	191.46	196.10	196.41	196.70	195.66	196.91

## Data Availability

The original contributions presented in the study are included in the article, further inquiries can be directed to the corresponding author.

## References

[1] Yuan G.M., Li Y., Long X.Y., Cui Z.W., Dong Z.J., Cong Y., Zhang J., Li X.K. (2020). Tuning anisotropic thermal conductivity of unidirectional carbon/carbon composites by incorporating carbonaceous fillers. J. Mater. Sci..

[2] Yang J., Ai Y., Lv X., Qi Z., Jiao J. (2021). Fabrication of C/C-SiC composites using high-char-yield resin. Int. J. Appl. Ceram. Technol..

[3] Zambrzycki M., Wielowski R., Gubernat M., Jantas D., Paczosa-Bator B., Fraczek-Szczypta A. (2024). The impact of chemical functionalization of carbon nanotubes on the electrochemical performance of carbon fiber/pyrocarbon/carbon nanotube composites as potential materials for electrodes for nerve cell stimulation. Appl. Surf. Sci..

[4] Marinković S., Dimitruević S. (1985). Carbon/carbon composites prepared by chemical vapour deposition. Carbon.

[5] Lee K.-J., Lee M.-C., Shih Y.-H., Lin H.-Y. (2023). Doping Effects of Carbon Nanotubes and Graphene on the Flexural Properties and Tribological Performance of Needle-Punched Carbon/Carbon Composites Prepared by Liquid-Phase Impregnation. Nanomaterials.

[6] Blanco C., Bermejo J., Marsh H., Menendez R. (1997). Chemical and physical properties of carbon as related to brake performance. Wear.

[7] Muhammed F., Lavaggi T., Advani S., Mirotznik M., Gillespie J.W. (2021). Influence of material and process parameters on microstructure evolution during the fabrication of carbon-carbon composites: A review. J. Mater. Sci..

[8] Zou J., Zeng X., Li X., Xiong X., Xie S., Qian H.J.M.T. (2010). Influence of gas partial pressure on density and texture of carbon/carbon composite fabrication by microwave pyrolysis chemical vapour infiltration. Mater. Technol..

[9] Yang W., Luo R.-Y., Hou Z.-H., Zhang Y., Shang H.-D., Hao M.-Y. (2016). Influence of the microstructure of the carbon matrices on the internal friction behavior of carbon/carbon composites. New Carbon Mater..

[10] Sinitsin A.N., Zuev V.V. (2017). Effect of fulleroid materials on the mechanical and tribological properties and dielectric relaxation of polyamide 6 nanocomposites. Polym. Adv. Technol..

[11] Yin X., Zhang X., Liu H., Fu Q., Li H. (2023). Novel Structural Design Strategies in Ceramic-Modified C/C Composites. Acc. Mater. Res..

[12] Shu J., Liao W., Zheng K., Abd Elbadia T. (2022). Effect of rotary ultrasonic machining on surface structure and ablation resistance of C/C composite. Ceram. Int..

[13] Valinejad Qanati M., Rasooli A. (2023). The effect of carbonization maximum temperature on the braking performance of the novalac-based carbon/carbon composite. Theor. Appl. Fract. Mech..

[14] Yang S., Bai S., Duan W., Wang Q. (2018). Engineering, Production of value-added composites from aluminum–plastic package waste via solid-state shear milling process. ACS Sustain. Chem. Eng..

[15] Hutton T.J., McEnaney B., Crelling J.C. (1999). Structural studies of wear debris from carbon-carbon composite aircraft brakes. Carbon.

[16] Windhorst T., Blount G. (1997). Carbon-carbon composites: A summary of recent developments and applications. Mater. Des..

[17] Feng S., Zhou X., Yang S., Tan J., Chen M., Chen Y., Zhang H., Zhu X., Wu S., Gu H.J.P. (2024). Preparation of Polyoxymethylene/Exfoliated Molybdenum Disulfide Nanocomposite through Solid-State Shear Milling. Polymers.

[18] Guo W., Liu Z., Yang S., Li L. (2023). Engineering, Fabrication of Sustainable Composite Foam from Ethylene Vinyl Acetate-Based Sole Waste via Solid-State Shear Milling and Supercritical Carbon Dioxide Foaming Technologies. ACS Sustain. Chem. Eng..

[19] Shao W., Wang Q., Wang F., Chen Y. (2006). The cutting of multi-walled carbon nanotubes and their strong interfacial interaction with polyamide 6 in the solid state. Carbon.

[20] Myalski J., Godzierz M., Olesik P.J.P. (2020). Effect of carbon fillers on the wear resistance of pa6 thermoplastic composites. Polymers.

[21] Chen J., Zhu J., Li Q., Wu H., Guo S., Qiu J. (2022). Constructing 3D interconnected CNTs network in PA6 composites with well-dispersed UHMWPE for excellent tribological and heat dissipation properties. Compos. Part B Eng..

[22] Ghanta T.S., Aparna S., Verma N., Purnima D. (2020). Review on nano-and microfiller-based polyamide 6 hybrid composite: Effect on mechanical properties and morphology. Polym. Eng. Sci..

[23] Kumar S.S., Kanagaraj G. (2016). Investigation on Mechanical and Tribological Behaviors of PA6 and Graphite-Reinforced PA6 Polymer Composites. Arab. J. Sci. Eng..

[24] Chen H., Cai Q., Wu J., Xia X., Liu H., Luo Z. (2018). Interfacial enhancement of carbon fiber/nylon 12 composites by grafting nylon 6 to the surface of carbon fiber. Appl. Surf. Sci..

[25] Zhang J., Du Z., Zou W., Li H., Zhang C. (2017). MgO nanoparticles-decorated carbon fibers hybrid for improving thermal conductive and electrical insulating properties of Nylon 6 composite. Compos. Sci. Technol..

[26] Man Z., Wang H., He Q., Kim D.-E., Chang L. (2021). Friction and wear behaviour of additively manufactured continuous carbon fibre reinforced PA6 composites. Compos. Part B Eng..

[27] Chen S., Cai L., Duan Y., Jing X., Zhang C., Xie F. (2024). Performance enhancement of 3D-printed carbon fiber-reinforced nylon 6 composites. Polym. Compos..

[28] Li Q., Rao R., Hong X., Hu H., Li Y., Gong Z., Zheng Y. (2024). Thermal conductive nylon 6 composites using hybrid fillers to construct a three-dimensional thermal conductive network. Polym. Compos..

[29] Liao Y., Chen C., Wei B., Yang S., Bai S. (2022). Green recycling of aramid fiber waste as the friction modifier of POM by solid state shear milling technology. Polym. Adv. Technol..

[30] (2010). Standard Test Method for Tensile Properties of Plastics.

[31] (2010). Standard Test Method for Tensile Properties of Plastics.

[32] (2010). Standard Test Method for Tensile Properties of Plastics.

[33] Wang S., Chen Z.-H., Ma W.-J., Ma Q.-S. (2006). Influence of heat treatment on physical–chemical properties of PAN-based carbon fiber. Ceram. Int..

[34] Xu X., Wang Q., Kong X., Zhang X., Huang J. (1996). Pan mill type equipment designed for polymer stress reactions: Theoretical analysis of structure and milling process of equipment. Plast. Rubber Compos. Process. Appl..

[35] Yang S., Zhong F., Wang M., Bai S., Wang Q. (2018). Recycling of automotive shredder residue by solid state shear milling technology. J. Ind. Eng. Chem..

[36] Wang X., Yang S., Wang Q. (2023). The experiment and simulation of enhanced mechanical performance for polyethylene terephthalate/high-density polyethylene composites via domain size control. Polym. Adv. Technol..

[37] Naito K. (2018). Stress analysis and fracture toughness of notched polyacrylonitrile (PAN)-based and pitch-based single carbon fibers. Carbon.

[38] Duan S., Liu F., Pettersson T., Creighton C., Asp L.E. (2020). Determination of transverse and shear moduli of single carbon fibres. Carbon.

[39] Chen H., Yu F., Wang B., Zhao C., Chen X., Nsengiyumva W., Zhong S. (2023). Elastic Fibre Prestressing Mechanics within a Polymeric Matrix Composite. Polymers.

[40] Venkateshwaran N., ElayaPerumal A., Alavudeen A., Thiruchitrambalam M. (2011). Mechanical and water absorption behaviour of banana/sisal reinforced hybrid composites. Mater. Des..

[41] Li W., He X., Zuo Y., Wang S., Wu Y. (2019). Study on the compatible interface of bamboo fiber/polylactic acid composites by in-situ solid phase grafting. Int. J. Biol. Macromol..

[42] Chen J., Zhu J., Wu H., Guo S., Qiu J. (2021). Constructing highly aligned crystalline structure to enhance sliding wear performance of bulk polyamide 6. Polymer.

[43] Jeong N., Cho D. (2023). Effect of Fiber Side-Feeding on Various Properties of Nickel-Coated Carbon-Fiber-Reinforced Polyamide 6 Composites Prepared by a Twin-Screw Extrusion Process. J. Compos. Sci..

[44] Hwang D., Cho D. (2019). Fiber aspect ratio effect on mechanical and thermal properties of carbon fiber/ABS composites via extrusion and long fiber thermoplastic processes. J. Ind. Eng. Chem..

